# *MTHFR* A1298C gene polymorphism on stroke risk: an updated meta-analysis

**DOI:** 10.1186/s41021-021-00208-z

**Published:** 2021-09-25

**Authors:** Xiaobo Dong, Jun Wang, Gesheng Wang, Jiayue Wang, Lei Wang, Yong Du

**Affiliations:** 1 The Third department of Encephalopathy, Dongfang Hospital Beijing University of Chinese Medicine, No. 6, Area 1, Fangxing Garden Fangzhuang, Fengtai District, Beijing, 100078 China; 2 Department of Neurology, Chinese PLA General Hospital, Beijing, 100039 China

**Keywords:** *MTHFR* A1298C, Polymorphism, Stroke, Meta-analysis

## Abstract

**Background:**

Previous studies have shown the effect of *MTHFR* A1298C gene polymorphism on stroke risk. But the results of published studies remained inconclusive and controversial. So we conducted a meta-analysis to accurately estimate the potential association between *MTHFR* A1298C gene polymorphism and stroke susceptibility.

**Methods:**

A systematic literature search on Embase, Pubmed, Web of Science, Cochrane Library, China National Knowledge Infrastructure (CNKI) and WanFang electronic database identified 40 articles including 5725 cases and 8655 controls. Strength of association was evaluated by pooled odds ratio (OR), 95% confidence interval (CI) and *p* value. Funnel plots and Begger’s regression test were applied for testing the publication bias. Statistical analysis of all data was performed by Stata 12.0.

**Results:**

The meta-analysis results indicated a significant relationship between *MTHFR* gene A1298C polymorphisms and stoke risk under the C allelic genetic model (OR = 1.19, 95%CI = 1.07–1.32, *p* = 0.001), dominant genetic model (OR = 1.19, 95%CI = 1.06–1.33, *p* = 0.004) and recessive genetic model (OR = 1.43, 95%CI =1.15–1.77, *p* = 0.001). In subgroup analysis, we discovered obvious correlation in three genetic model of Asian, stroke type, adult by ethnicity, population, stroke type, source of control and case size. Additionally, in studies of control from hospital and case size equal 100, obvious correlation was also found in the three genetic model.

**Conclusions:**

Our meta-analysis results indicated that there was evidence to support the correlation between M*THFR* A1298C polymorphism and stroke susceptibility, especially in adults and ischemic stroke.

## Introduction

Stroke was a type of clinical syndrome caused by sudden neurological deficits after cerebral blood vessel rupture or occlusion. It had a very high rate of disability and was classified into ischemic stroke (IS) and hemorrhagic stroke (HS) [1, 2]. Genetic genes, high blood sugar, unhealthy lifestyles, high blood pressure, and hyperlipidemia were all high-risk factors for stroke, which similarly to high-risk factors for cardiovascular disease [3, 4]. Methylenetrahydrofolate reductase (MTHFR) was a key enzyme that folic acid metabolizes in vivo. The activity of this enzyme can directly affect the plasma homocysteine content in the human body [5, 6]. C677T and A1298C were two common mutants in *MTHFR*. Their missense mutations resulted in the replacement of 677 base C with T and the substitution of A with C in 1298, which changed the amino acid structure of MTHFR and caused the decrease of MTHFR enzyme activity [7–10]. The homocysteine cannot be converted into the word methionine normally, which causes a significant increase in the homocysteine content in the blood, which increases the stroke susceptibility [11].

A meta-analysis study performed in 2013 firstly reported the association among *MTHFR* A1298C and stroke risk [12]. A meta-analysis study performed in 2014 reported the correlation between *MTHFR* A1298C polymorphism and adult stroke [13]. Although Kumar et al. [14] conducted a meta- analysis and indicated that genotyping of *MTHFR* gene A1298C polymorphism may be used as a predictor for the occurrence of ischemic stroke. However, just 20 articles were included in this study, and some articles published in Chinese journal were not included in the analysis. Incomplete article search may lead to unstable results. Later a meta-analysis study performed in 2018 reported the association among *MTHFR* A1298C polymorphism and IS risk [15]. In recent years, many studies [16–20] have reported the association among *MTHFR* A1298C polymorphism and stroke risk. But the results were still inconsistent. Therefore, the purpose of this meta-analysis was to investigate the relationship among *MTHFR* A1298C polymorphism and stroke risk by updating previous meta-analyses.

## Materials and methods

### Publication search

Search in electronic databases such as PubMed, Cochrane Library, Web of Science, Embase, CNKI and WanFang using following terms: (“methylenetetrahydrofolate reductase” OR “A1298C*”* OR “*MTHFR*”) AND (“apoplexy” OR “stroke” OR “brain infarction” OR “cerebrovascular disorder”) AND (“polymorphism” OR “variant” OR “mutation”) until August 2019. To ensure the relevant studies are included, two investigators independently searched the relevant literature and manually checked some major articles and reviews.

### Selection Criteria

ALL selected studies complied with the inclusion criteria: (1) Full text can be searched in electronic databases; (2) Case-control studies on *MTHFR* A1298C and stroke susceptibility; (3) *MTHFR* A1298C genotype frequency can be provided. The main exclusion criteria include the following: (1) Repeat articles in other electronic databases (2) The design was not a case-control study; (3) Unpublished studies, meta- analysis and systematic reviews; (4) The genotype frequency of *MTHFR* A1298C was not provided. Referring to the systematic review and meta-analysis [PRISMA], we screened all retrieved documents and constructed an information flow chart using the final qualified data.

### Data extraction

Two researchers screened the retrieved studies by inclusion and exclusion standard. We selected following information from the included researches: first author, publication years, study country / region, type of stroke, study population, control group source, sample size (case and control) and genotype type. we evaluated each included study by Newcastle-Ottawa Scale (NOS) [21]. And we used Hardy-Weinberg-equilibrium (HWE) to assess the gene distribution in control group [22]. In order to ensure the accuracy of the information extracted from the research, the third researcher will review the accuracy of the information extracted by the first two researchers, and the three researchers will resolve the disputed results through negotiation.

### Statistical analysis

Odds ratio (OR) and 95% confidence interval (CI) were calculated to estimate the relationship among *MTHFR* A1298C polymorphism and stroke susceptibility by different genetic comparison models: the C allele model (C and A), recessive model (CC and AA + CA) and dominant model (CC + CA and AA). Heterogeneities of different genetic comparison models was evaluated by the χ^2^ based Q-statistic and *I*^2^ [23, 24]. Significant Q statistic (*p* < 0.10) or *I*^2^ > 50% indicates that there was heterogeneity between studies. The pooled OR was estimated by fixed effect model (Mantel–Haenszel) when no heterogeneity existed. Otherwise, the pooled OR was estimated by random effect model. To determine the possible causes of heterogeneity, ethnicity (African, Caucasian and Asian), study population (children and adults), type of stroke (ischemic and hemorrhagic), control source (based on population and Hospitals) and case group sample sizes (less than 100 and greater than or equal to 100) were analyzed in subgroups. In addition, sensitivity analysis was performed on different genetic comparison models to evaluate the effect of a single research on pooled OR. We used Egger test and funnel plots to evaluate the potential publication bias [25]. Stata 12.0 was used to perform statistical analysis of all genetic comparison models.

## Results

### Literature Search and Study Characteristics

**Fig. 1:**
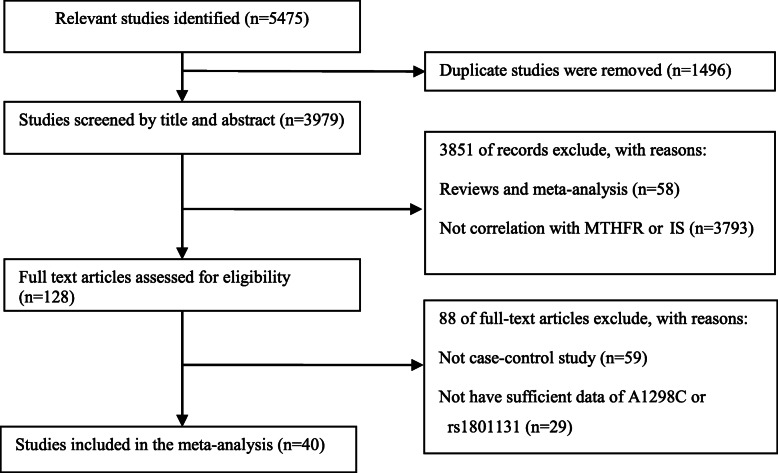
The flow sheet of identification of eligible studies

Flowcharts of the detailed selection process were shown in Fig. 1. A total of 5475 publications were searched in several electronic databases. After carefully reading the research title, content and abstract, the two researchers excluded 1496 duplicate documents, 3851 irrelevant papers, and read the remaining 128 articles in full text. Finally, our meta-analysis included 40 case-control publications that met the inclusion criteria (involving 5725 cases and 8655 controls). Four of the control groups did not meet HWE balance (*p* < 0.05). In the 40 case-control studies, 22 were conducted among Asian populations, 16 were Caucasian, and 2 were African population. Thirty-one studies were based on adults. Other 9 studies were based on children. Ten studies were based on IS. Four studies based on HS and other studies based on both (MIXED). Four studies based on population (PB), 21 studies based on hospital (HB). other studies based on no report (NR) in this article. The sample group of 21 studies was less than 100, and the sample group of the other 19 studies was greater than or equal to 100. The main features of the study and the genotype distribution results of the HWE test were shown in Table 1.

**Table 1:** Studies and data included in this meta-analysis

Author	Year	Country	Type	Population	Source of control	Sample size		case					control					NOS score	HWE
						case	control	CC	CA	AA	C	A	CC	CA	AA	C	A		
Akar et al	2001	Turkey	IS	Children	NR	46	68	13	27	6	53	39	31	32	5	94	42	7	0.399
Lin et al	2004	China	IS	Adults	HB	68	50	50	16	2	116	20	35	15	0	85	15	8	0.212
Linnebank et al	2005	Germany	IS	Adults	PB	159	159	80	65	14	225	93	73	64	22	210	108	8	0.196
Sun et al	2005	China	IS	Adults	NR	97	94	55	40	2	150	44	60	31	3	151	37	8	0.676
Komitopoulou et al	2006	Greece	IS	Children	HB	89	103	35	45	9	115	63	50	40	13	140	66	7	0.272
Sazci et al	2006	Turkey	IS	Adults	NR	92	259	36	37	19	109	75	130	108	21	368	150	8	0.828
Sazci et al	2006	Turkey	HS	Adults	NR	28	259	16	9	3	41	15	130	108	21	368	150	8	0.828
Dikmen et al	2006	Turkey	IS	Adults	NR	154	55	59	79	16	197	111	19	33	3	71	39	7	0.021
Dikmen et al	2006	Turkey	HS	Adults	NR	49	55	19	23	7	61	37	19	33	3	71	39	7	0.021
Zhang et al	2007	China	IS	Adults	NR	100	100	56	40	4	152	48	64	33	3	161	39	8	0.609
Sirachainan et al	2008	Thailand	IS	Children	HB	44	164	22	19	3	63	25	82	69	13	233	95	6	0.774
Biswas et al	2009	India	IS	Children	NR	58	58	38	14	6	90	26	50	7	1	107	9	8	0.232
Morita et al	2009	America	IS	Children	PB	23	90	17	6	0	40	6	41	36	13	118	62	6	0.278
Sawula et al	2009	Poland	IS	Adults	HB	128	59	53	57	18	163	93	26	22	11	74	44	7	0.119
Almawi et al	2009	Bahrain	MIXED	Adults	HB	118	120	50	38	30	138	98	54	60	6	168	72	6	0.037
Biswas et al	2009	India	IS	Adults	HB	120	120	80	31	9	191	49	90	24	6	204	36	7	0.018
Chen et al	2010	China	IS	Adults	HB	470	495	354	109	7	817	123	387	105	3	879	111	7	0.146
Han et al	2010	Korea	IS	Adults	HB	264	234	179	80	5	438	90	182	51	1	415	53	8	0.193
Giusti et al	2010	Italy	IS	Adults	HB	501	1211	248	197	56	693	309	572	529	110	1673	749	7	0.434
Hultdin et al	2011	Sweden	IS	Adults	NR	314	767	135	142	37	412	216	328	346	93	1002	532	7	0.905
Hultdin et al	2011	Sweden	HS	Adults	NR	59	767	29	29	1	87	31	328	346	93	1002	532	7	0.905
Arsene et al	2011	Bahrain	IS	Adults	HB	67	60	30	28	9	88	46	25	27	8	77	43	7	0.868
Zhang et al	2012	China	IS	Adults	HB	67	71	38	29	0	105	29	57	14	0	128	14	7	0.357
Xu et al	2012	China	MIXED	Adults	HB	70	50	7	41	22	55	85	12	30	8	54	46	8	0.142
Zhang et al	2012	China	IS	Adults	HB	40	40	27	12	1	66	14	33	7	0	73	7	8	0.544
Fekih-Mrissa et al	2013	Tunisia	IS	Adults	HB	84	100	58	15	11	131	37	93	7	0	193	7	7	0.717
Gelfand et al	2013	America	IS	Children	PB	13	84	4	7	2	15	11	39	41	4	119	49	8	0.097
Zhou et al	2014	China	IS	Adults	HB	542	654	333	174	35	840	244	448	182	24	1078	230	8	0.308
Balcerzyk et al	2015	Poland	IS	Children	NR	88	111	51	30	7	132	44	53	47	11	153	69	7	0.902
Lv et al	2015	China	IS	Adults	NR	199	241	31	97	71	159	239	71	110	60	252	230	8	0.186
Wei et al	2015	Malaysia	IS	Adults	NR	297	297	184	95	18	463	131	186	104	7	476	118	6	0.085
Kamberi et al	2016	Albania	IS	Adults	PB	39	102	18	20	1	56	22	54	44	4	152	52	8	0.171
Herak et al	2017	Croatia	IS	Children	HB	73	100	34	33	6	101	45	52	39	9	143	57	8	0.667
Wang et al	2017	China	MIXED	Adults	NR	225	169	130	85	10	345	105	99	65	5	263	75	6	0.139
Zhao et al	2017	China	IS	Adults	HB	130	100	98	32	0	228	32	68	32	0	168	32	8	0.057
Hu et al	2017	China	IS	Adults	HB	181	169	106	67	8	279	83	99	65	5	263	75	8	0.139
Abidi et al	2018	Morocco	HS	Adults	HB	113	323	64	46	3	174	52	186	120	17	492	154	8	0.678
Hashemi et al	2019	Iran	IS	Adults	NR	106	157	72	31	3	175	37	120	32	5	272	42	8	0.131
Xiong et al	2019	China	MIXED	Adults	HB	92	140	64	24	4	152	32	90	44	6	224	56	8	0.833
Mazdeh et al	2020	Iran	IS	Children	HB	318	400	170	121	27	461	175	235	147	18	617	183	8	0.406

### Meta-analysis results

The meta-analysis included 14,380 participants (5725 cases and 8655 controls) in 40 case-control studies (Table 2). The meta-analysis results indicated the polymorphisms in *MTHFR* A1298C gene had significant association with stoke risk under the C allelic genetic model (OR = 1.19, 95%CI = 1.07–1.32, *p* = 0.001), dominant genetic model (OR = 1.19, 95%CI = 1.06–1.33, *p* = 0.004) and recessive genetic model (OR = 1.43, 95%CI =1.15–1.77, *p* = 0.001).

**Table 2:** Pooled ORs and 95%CIs of the association between *MTHFR* A1298C polymorphism and stroke

Total and subgroups	Studies	CC + CA vs AA				CC vs CA + AA				C VS A			
		OR	95%CI	I^2^	P	OR	95%CI	I^2^	P	OR	95%CI	I^2^	P
Total	40	1.19	1.06 ~ 1.33	51.8%	< 0.001	1.43	1.15 ~ 1.77	40.6%	0.006	1.19	1.07 ~ 1.32	61.1%	< 0.001
Ethnicity													
Asian	22	1.28	1.17 ~ 1.47	40.9%	0.025	1.84	1.49 ~ 2.27	0.0%	0.057	1.29	1.16 ~ 1.44	36.8%	0.044
Caussian	16	0.99	0.85–1.17	29.2%	0.131	1.10	0.81 ~ 1.50	44.0%	0.031	1.01	0.88 ~ 1.16	48.0%	0.017
African	2	2.38	0.43–13.19	91.6%	0.001	3.31	0.04 ~ 276.07	87.8%	0.004	2.63	0.33 ~ 20.97	95.2%	< 0.001
Population													
Child	9	1.20	0.85 ~ 1.69	58.9%	0.013	1.25	0.79 ~ 2.00	29.0%	0.187	1.15	0.86 ~ 1.54	66.1%	0..003
Adult	31	1.19	1.06 ~ 1.34	51.1%	0.001	1.48	1.16 ~ 1.89	45.0%	0.005	1.18	1.07 ~ 1.32	60.9%	< 0.001
Stroke type													
IS	32	1.24	1.09 ~ 1.42	56.6%	< 0.001	1.38	1.12 ~ 1.69	28.4%	0.076	1.22	1.09 ~ 1.37	63.0%	< 0.001
HS	4	0.89	0.67 ~ 1.18	0.0%	0.811	0.79	0.23 ~ 2.65	64.5%	0.037	0.88	0.70 ~ 1.10	0.0%	0.494
MIXED	4	1.09	0.77 ~ 1.56	36.8%	0.191	2.39	1.09 ~ 5.22	55.9%	0.078	1.27	0.91 ~ 1.78	60.2%	0.057
Source of control													
HB	21	1.23	1.06 ~ 1.44	51.4%	0.011	1.54	1.28 ~ 1.86	40.9%	0.033	1.24	1.08 ~ 1.42	60.7%	< 0.001
PB	4	0.88	0.47 ~ 1.62	52.7%	0.003	0.61	0.33 ~ 1.11	39.5%	0.175	0.86	0.49 ~ 1.50	68.8%	0.022
NR	15	1.19	0.98 ~ 1.44	57.9%	0.068	1.38	1.12 ~ 1.70	37.4%	0.071	1.18	1.01 ~ 1.39	59.2%	0.002
Case size													
< 100	21	1.28	1.00 ~ 1.65	61.3%	< 0.001	1.36	0.92 ~ 2.01	36.9%	0.050	1.25	1.00 ~ 1.57	69.8%	< 0.001
≥100	19	1.14	1.02 ~ 1.28	36.8%	0.055	1.46	1.12 ~ 1.88	47.5%	0.013	1.16	1.05 ~ 1.28	46.8%	0.013

**Fig. 2:**
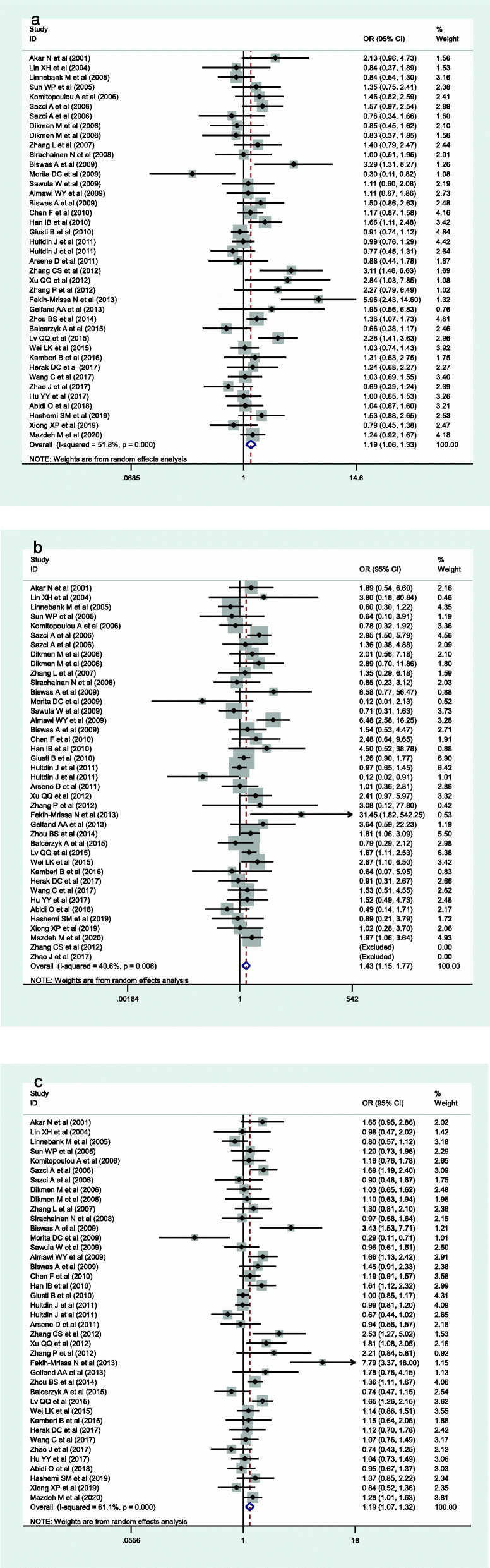
Forest plots of the *MTHFR* A1298C polymorphism under different genetic models. **a** is the model of CC + CA VS TT; **b** is the model of CC VS CA + AA; **c** is the model of C VS A

In ethnic subgroup analysis, *MTHFR* A1298C polymorphism was obviously correlated with increased risk of stroke under three genetic models of Asian population (C vs A: OR = 1.29, 95%CI = 1.16–1.44, *p* < 0.001; CC + CA vs AA: OR = 1.28, 95%CI = 1.17–1.47, *p* < 0.001; CC vs CA + AA: OR = 1.84, 95%CI = 1.49–2.27, *p* < 0.001). No significant correlation among *MTHFR* A1298C Polymorphism and stroke risk was found in the three genetic models of Caucasian and African. Among the three genetic models grouped by study population, only adult *MTHFR* A1298C polymorphisms were found to be obviously correlated with stroke susceptibility (C vs A: OR = 1.18, 95%CI = 1.07–1.32, *p* = 0.002; CC + CA vs AA: OR = 1.19, 95%CI = 1.06–1.34, *p* = 0.008; CC vs CA + AA: OR = 1.48, 95%CI = 1.16–1.89, *p* = 0.002). There was no obvious association among *MTHFR* A1298C polymorphism and children stroke risk. Stratified analysis by stroke type found that *MTHFR* A1298C polymorphism was obviously correlated with increased stroke risk in the three genetic models of ischemic stroke (C vs A: OR = 1.22, 95%CI = 1.09–1.37, *p* = 0.002; CC + CA vs AA: OR = 1.24, 95%CI = 1.09–1.42, *p* = 0.002; CC vs CA + AA: OR = 1.38, 95%CI = 1.12–1.69, *p* = 0.002). In the control source stratification, three control genetic models from hospitals found an obvious correlation among the *MTHFR* A1298C polymorphism and increased stroke risk (C vs A: OR = 1.24, 95%CI = 1.08–1.42, *p* = 0.002; CC + CA vs AA: OR = 1.23, 95%CI = 1.06–1.44, *p* = 0.008; CC vs CA + AA: OR = 1.54, 95%CI = 1.28–1.86, *p* = 0.007). Finally, we stratified the case group according to whether the sample size was greater than or equal to 100. The study discovered that *MTHFR* A1298C polymorphism was obviously correlated with increased stroke risk under the three genetic models with a sample size of 100 or more (C vs A: OR = 1.16, 95%CI = 1.05–1.28, *p* = 0.003; CC + CA vs AA: OR = 1.14, 95%CI = 1.02–1.28, *p* = 0.020; CC vs CA + AA: OR = 1.46, 95%CI = 1.12–1.88, *p* = 0.004) (Fig. 2).

**Fig. 3:**
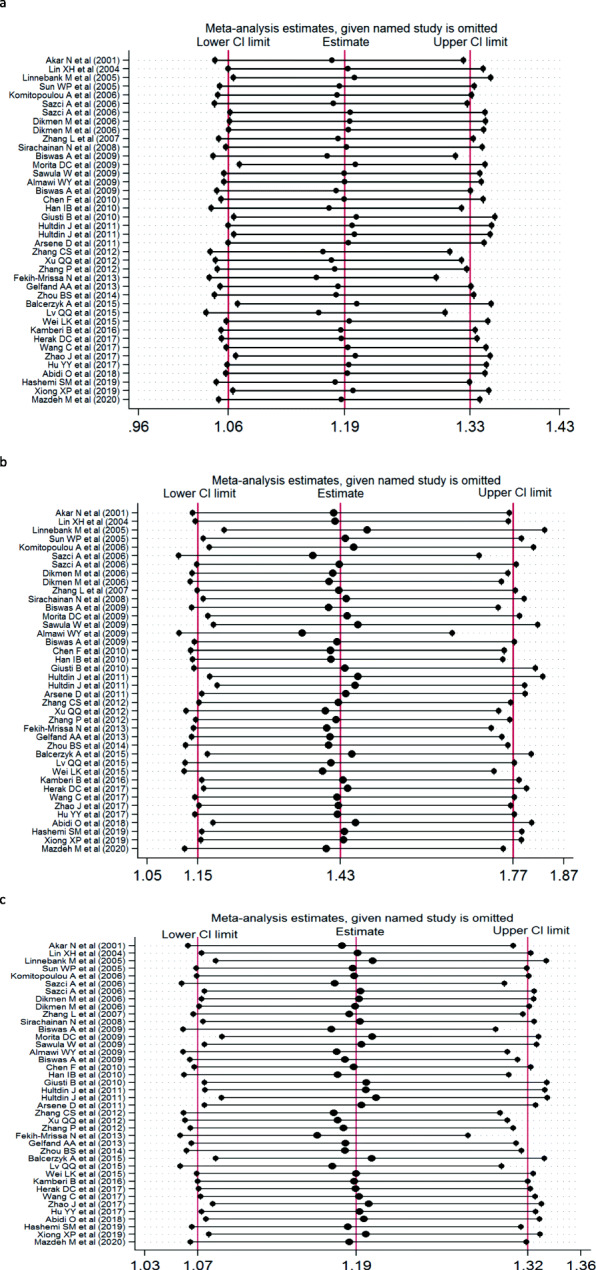
Sensitivity analysis examining the association between the *MTHFR* A1298C polymorphism and risk of stroke under these model (CC + CA VS AA, CC VS CA + AA, C VS A

**Fig. 4:**
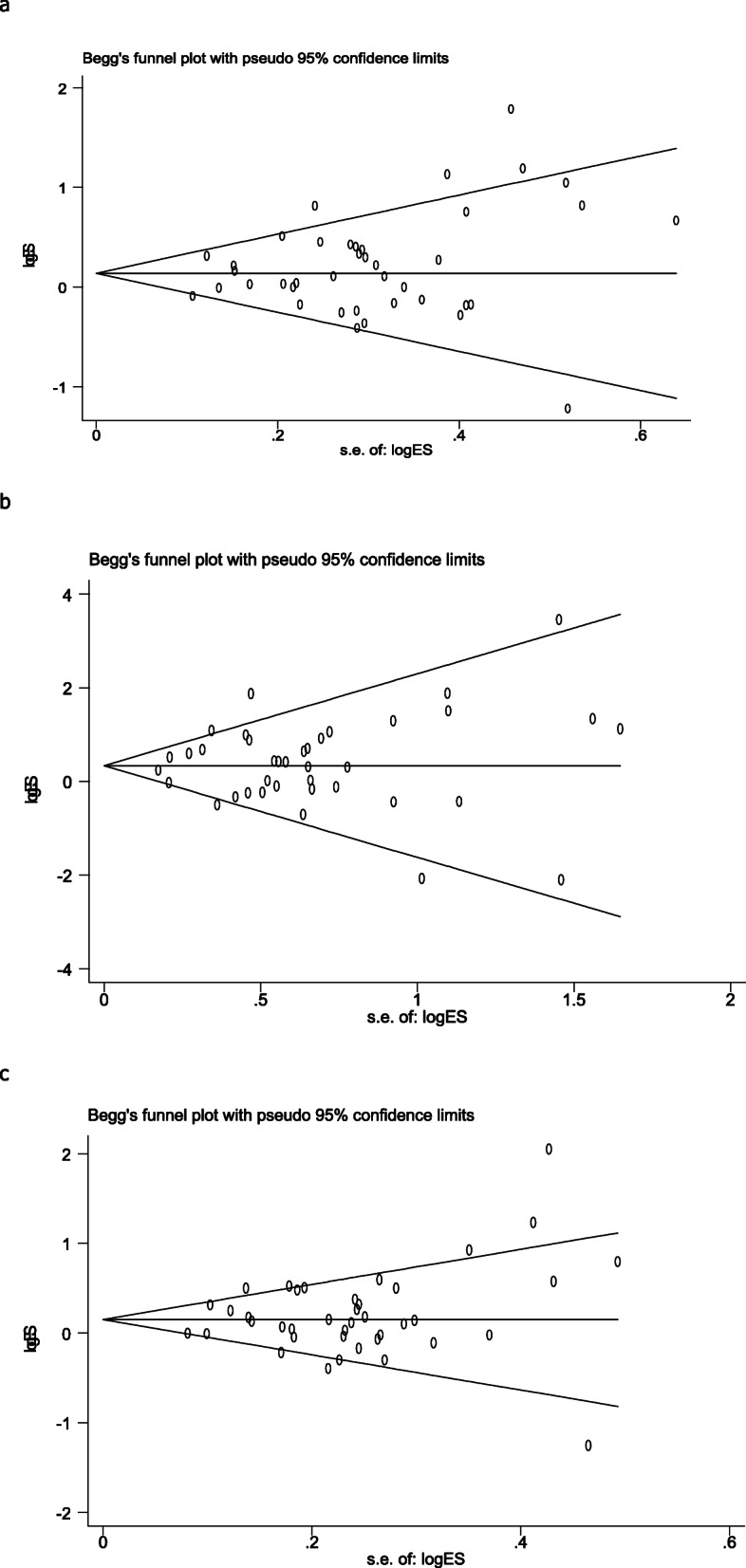
Begg’s funnel plot for publication bias analysis. **a** is the model of CC + CA VS TT; **b** is the model of CC VS CA + AA; c is the model of C VS A

In order to evaluate the stability of this meta-analysis, the sensitivity analysis of this study excluded each included study one by one to compare the difference between the pooled OR before and after exclusion. The results of this analysis were very stable (Fig. 3). We used Begg’ s funnel plot to estimate publication bias and found no publication in the three genetic models (C vs A: *p* = 0.742; CC + CA vs AA: *p* = 0.825; CC vs CA + AA: *p* = 0.138) (Fig. 4).

## Discussion

In recent years, there have been many studies on *MTHFR* A1298C polymorphism and stroke susceptibility [17, 19, 26–50]. In 2001, Akar et al. firstly found no association among *MTHFR* A1298C polymorphism and the ischemic stroke risk in Turkish children [51]. In 2004, the study by Lin et al. focused on adult ischemic stroke in China and found that the CC genotype and C allele frequency had no obvious difference between the cases and controls [52]. Biswas et al. [53] found that *MTHFR* 1298 A > C showed significant alleles and genotypes associated with disease phenotypes in the Indian child population. With increasing research among *MTHFR* A1298C polymorphism and stroke susceptibility, Lv et al. [12] performed a meta-analysis of *MTHFR* gene A1298C and stroke. In this meta-analysis, 13 studies with 1974 cases and 2660 controls were extracted to assess the potential correlation. Overall analysis indicated that *MTHFR* A1298C was significantly associated with the stroke risk only in the heterozygote comparison and in the dominant model. Zhang constructed a meta-analysis of *MTHFR* A1298C polymorphism and stroke risk in adults [13]. 15 researches with 2361 cases and 2653 controls were included in final meta-analysis. Comprehensive analysis results showed that the polymorphism of *MTHFR* gene A1298C was significantly correlated with adult stroke in allelic model, dominant, additive and recessive models. Because the two meta-analyses came from different populations and sample sizes are different, these studies have shown inconsistent results. Since 2014, there have been another 25 studies on *MTHFR* gene A1298C polymorphism and stroke. Therefore, we upgraded a meta-analysis of *MTHFR* gene A1298C polymorphism and stroke susceptibility.

This meta-analysis resolved the correlation among *MTHFR* A1298C polymorphism and stroke susceptibility. The comprehensive data of the study showed that *MTHFR* A1298C polymorphism was a probable risk factor for stroke in dominant model (CC + CA vs AA), recessive model (CC vs CA + AA) and allele model (C vs A). In stratified analysis based on race, study population, stroke type, source of controls population and sample size of cases, a significant association was discovered among *MTHFR* A1298C polymorphism and stroke in three genetic models of Asians. In Caussian and African, *MTHFR* A1298C polymorphism was not significantly correlated with stroke. In stratified analysis according to study population, it was discovered that *MTHFR* A1298C polymorphism was obviously correlated with stroke in adults. But the correlation between *MTHFR* A1298C polymorphism and stroke in children lacked corresponding evidence. In stratified analysis of stroke types, the association among *MTHFR* A1298C polymorphism and stroke was found only in ischemic stroke. The stratified analysis of source of the control group showed that there was obvious correlation among *MTHFR* A1298C polymorphism and stroke in hospital study. The stratified analysis of the sample size showed that the correlation among *MTHFR* A1298C polymorphism and stroke was found only when the number of samples in the case group was greater than or equal to 100. The above analysis showed that the source of control group and the sample size of case group may be the influencing factors of the correlation study among *MTHFR* A1298C polymorphism and stroke. This was undiscovered in early meta-analysis. Although Kumar et al. [14] conducted a meta- analysis on association between A1298C polymorphism and risk of ischemic stroke. However, just 20 articles were included in this study, and some articles published in Chinese journal were not included in the analysis. The biological mechanism of the association between A1298C and stroke has not been confirmed. Study [54] indicated that *MTHFR* gene can encode MTHFR enzyme, which plays a key role in regulating cellular homocysteine (Hcy) and folate metabolism by catalyzing the conversion of 5,10-methylpentylenetetrahydrofolate to 5- methyltetrahydrofolate, and elevated homocysteine level in blood circulation is considered as an independent risk factor for cerebral, coronary and peripheral atherosclerosis [55].

This meta-analysis has several limitations. Our results show the genetic differences in ethnic differences and stroke risk, but the study only includes Asian, Caucasian and African populations, and there are few studies in African populations, and there are no corresponding studies for other ethnic populations. The occurrence of stroke is often caused by the interaction of genetic factors and environmental factors. This study is only conducted from the perspective of genetics without the influence of environmental exposure. In previous studies, especially in meta-analysis, the data was still insufficient. We have checked as many articles as possible, but many studies have omitted data, such as control sources and genetic testing methods.

In conclusion, we found obvious correlation among *MTHFR* A1298C and stroke risk in Asians, adults and ischemic strokes. However, for the Caucasian, African, children and hemorrhagic stroke, the risk of *MTHFR* A1298C could not be confirmed because of the relatively limited sample size. In addition, sample size of case group and source of control group would also have an impact on the results in the stratified analysis of this study. Therefore, in future research, we can explore more about the correlation among *MTHFR* A1298C and stroke in other races (except for Asian population), children, and hemorrhagic stroke.

## Data Availability

Not applicable.
